# Supplementary material for: 3D PCL/Gelatin/Genipin Nanofiber Sponge as Scaffold for Regenerative Medicine
Source: Materials (Basel). 2021 Apr 16;14(8):2006. doi: 10.3390/ma14082006 (PMC8072632; doi:10.3390/ma14082006)
Supplement: Supplementary file 1 [file materials-14-02006-s001.zip › materials-1171509-supplementary.pdf]

Supplementary Information for

3D PCL/Gelatin/Genipin Nanofiber Sponge as Scaffold for Regenerative Medicine

Markus Merk[1,2], Orlando Chirikian[2] and Christian Adlhart[1*]

[1]Institute of Chemistry and Biotechnology, Zurich University of Applied Sciences ZHAW, 8820 Wädenswil, Switzerland. [2]Biomolecular Science and Engineering, University of California Santa Barbara UCSB, Santa Barbara, CA 93106, USA.

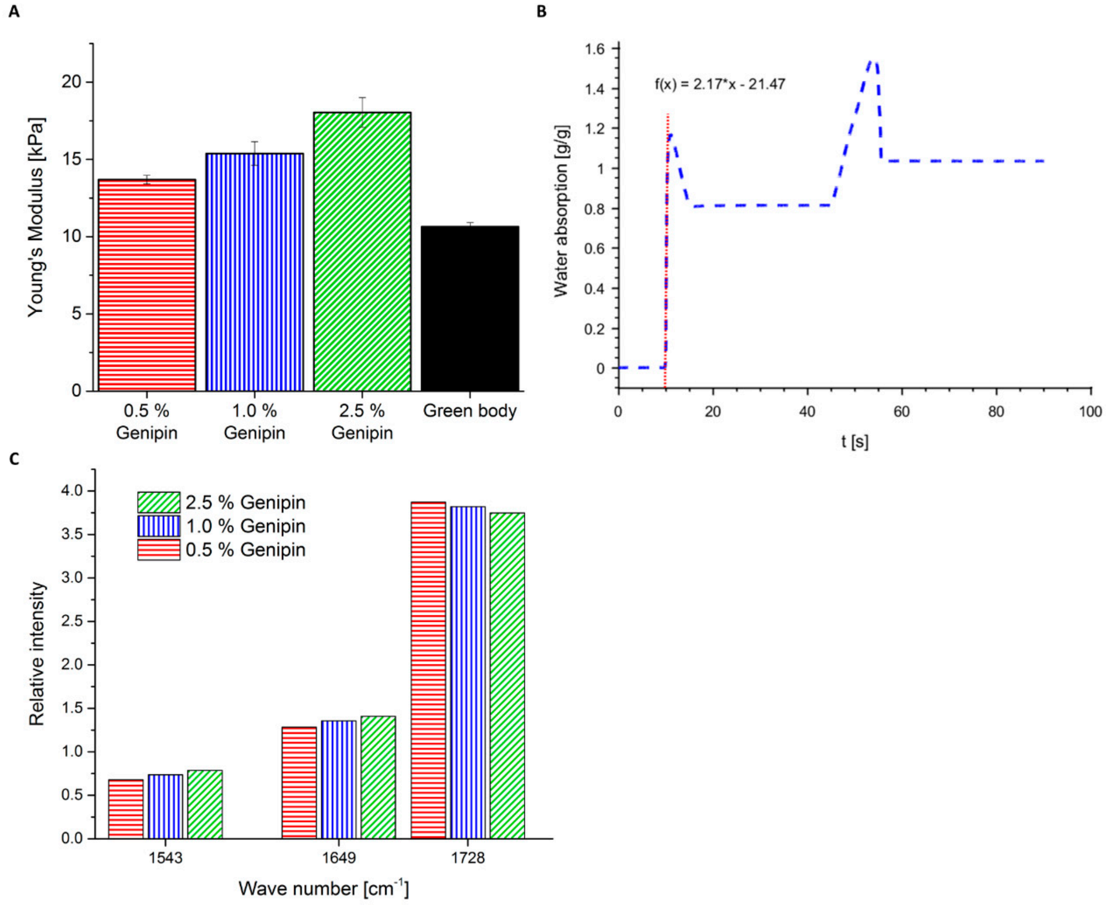

Figure S1: Characterization of different 3D scaffolds. (A) Young's moduli of 3D PCL/gelatin scaffolds cross-linked with either 0.5 %, 1.0 % or 2.5 % genipin and before cross-linking (green body). (B) Representative plot of the water absorption analysis, showing a scaffold cross-linked with 1 % genipin. Red dotted line indicates the applied linear regression for the water absorption rate measurements. (C) FTIR quantification of the cross-linked scaffolds. Wave number 1543 cm^{-1} and 1649 cm^{-1} correspond to the amide I and II bonds, respectively, and wave number 1728 cm^{-1} corresponds to an ester bond.

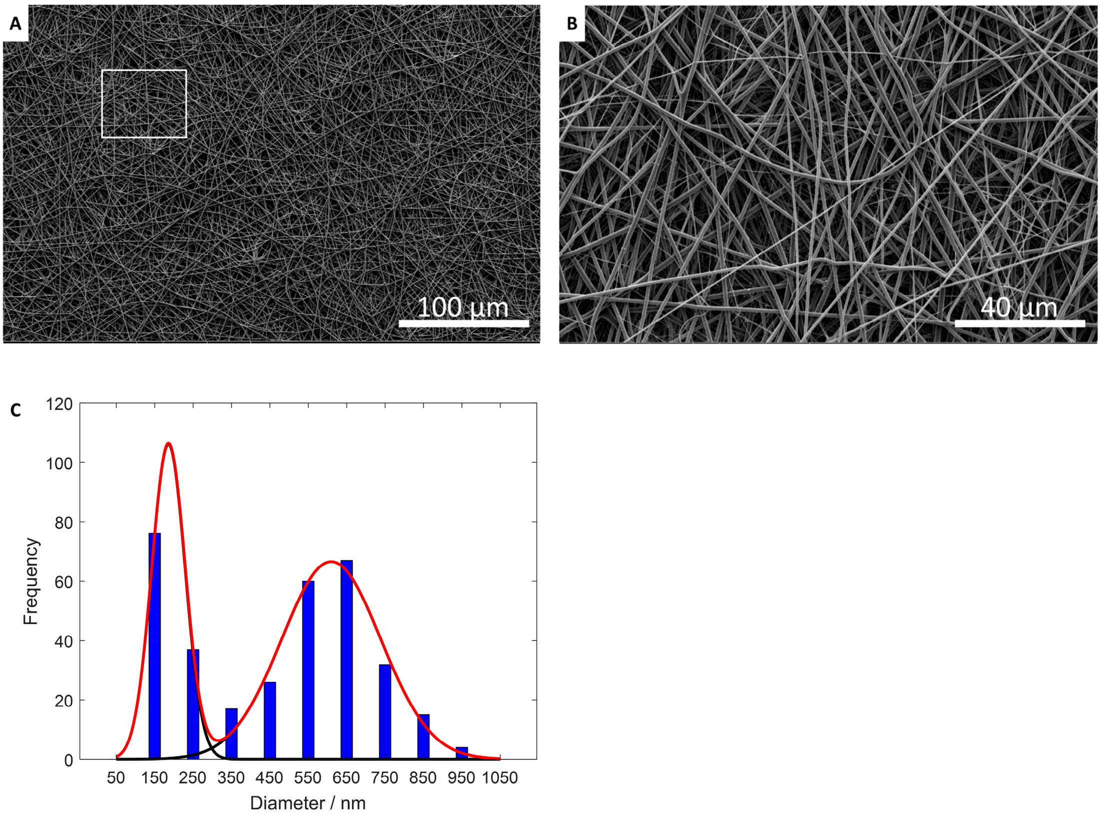

Figure S2: SEM image of electrospun 2D PCL/gelatin nanofiber mat (A) Composite fibers with a PCL to gelatin ratio of 2:1, scale bar = 100 µm. (B) Magnification of white insert in figure A, scale bar = 40 µm. (C) Fiber diameter distribution of electrospun nanofibers.

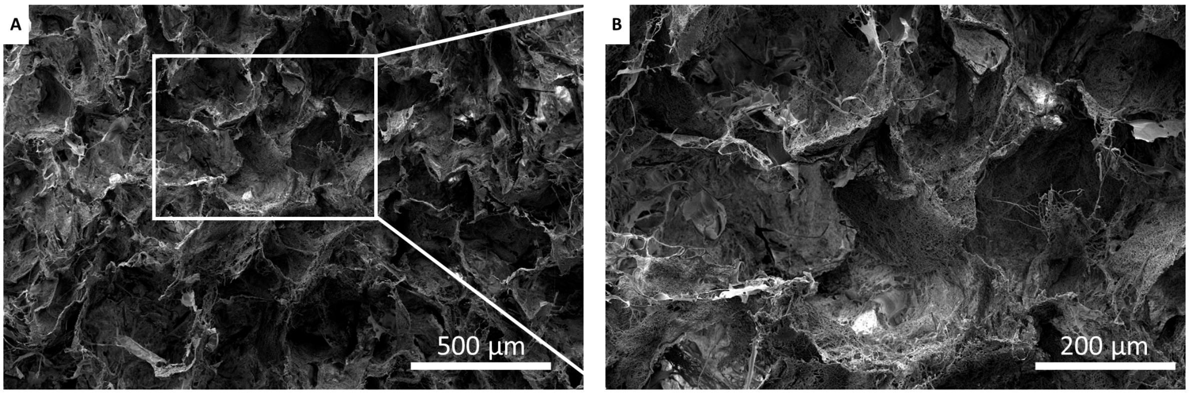

Figure S3: SEM image of 3D PCL/gelatin scaffolds. (A) Samples before cross-linking with genipin (green body), scale bar = 500 µm. (B) Magnification of white insert in figure A, scale bar = 200 µm.

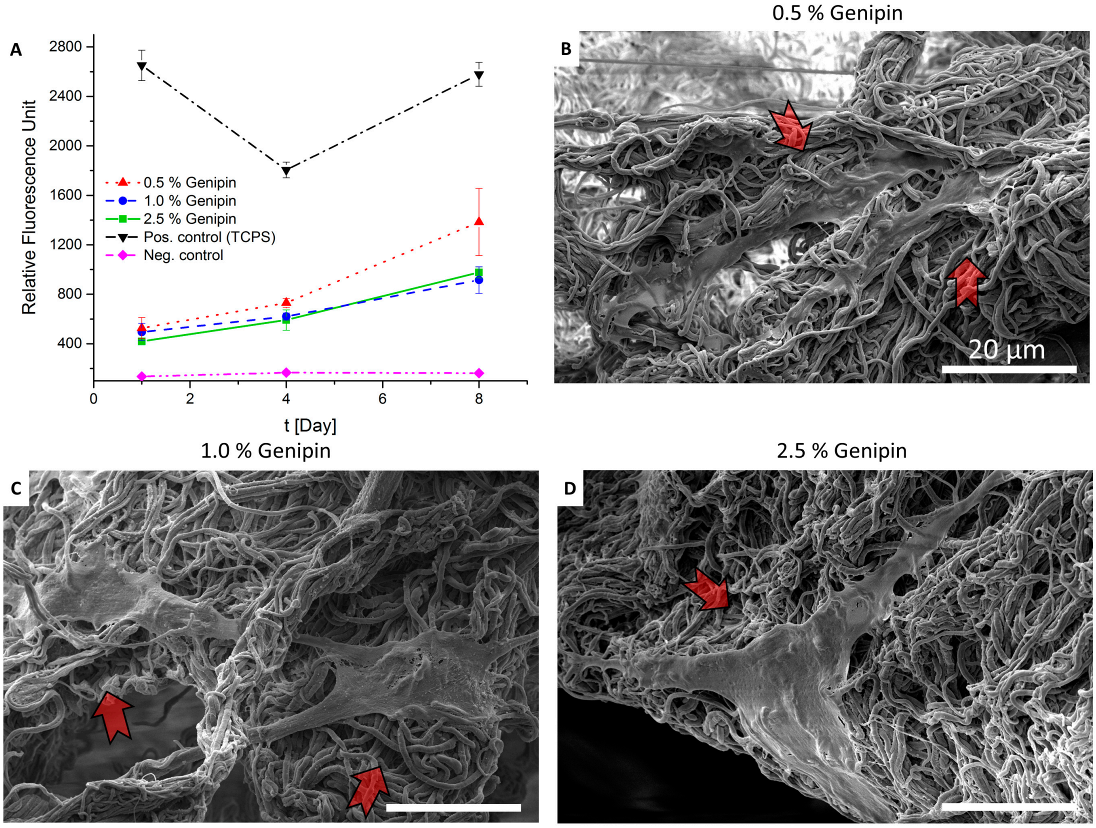

Figure S4: Short-term in vitro cell compatibility study using human dermal fibroblasts (HDF). (A) Metabolic activity measurements using the PrestoBlue assay. (B-D) SEM image of the cultured scaffolds after 8 days in culture. Red arrows indicate adhering and proliferating HDFs. (B) Scaffolds cross-linked with 0.5 % genipin. (C) Scaffolds cross-linked with 1.0 % genipin. (D) Scaffolds cross-linked with 2.5 % genipin. (B-D) Scale bar = 20 µm.
